# Interobserver reliability of scapula fracture classifications in intra- and extra-articular injury patterns

**DOI:** 10.1186/s12891-022-05146-7

**Published:** 2022-03-01

**Authors:** Henner Huflage, Tabea Fieber, Christian Färber, Jonas Knarr, Simon Veldhoen, Martin C. Jordan, Fabian Gilbert, Thorsten Alexander Bley, Rainer H. Meffert, Jan-Peter Grunz, Jonas Schmalzl

**Affiliations:** 1 Department of Diagnostic and Interventional Radiology, University Hospital Würzburg, Oberdürrbacher Straße 6, 97080 Würzburg, Germany; 2 Department of Trauma-, Hand-, Plastic- and Reconstructive Surgery, University Hospital Würzburg, Oberdürrbacher Straße 6, 97080 Würzburg, Germany; 3 Department of Orthopaedics and Trauma Surgery, University Hospital, LMU Munich, Ziemssenstraße 5, 80336 Munich, Germany

**Keywords:** Scapula, Glenoid, Fracture, Classification, Reliability, Confidence

## Abstract

**Background:**

Morphology and glenoid involvement determine the necessity of surgical management in scapula fractures. While being present in only a small share of patients with shoulder trauma, numerous classification systems have been in use over the years for categorization of scapula fractures. The purpose of this study was to evaluate the established AO/OTA classification in comparison to the classification system of Euler and Rüedi (ER) with regard to interobserver reliability and confidence in clinical practice.

**Methods:**

Based on CT imaging, 149 patients with scapula fractures were retrospectively categorized by two trauma surgeons and two radiologists using the classification systems of ER and AO/OTA. To measure the interrater reliability, Fleiss kappa (κ) was calculated independently for both fracture classifications. Rater confidence was stated subjectively on a five-point scale and compared with Wilcoxon signed rank tests. Additionally, we computed the intraclass correlation coefficient (ICC) based on absolute agreement in a two-way random effects model to assess the diagnostic confidence agreement between observers.

**Results:**

In scapula fractures involving the glenoid fossa, interrater reliability was substantial (κ = 0.722; 95% confidence interval [CI] 0.676–0.769) for the AO/OTA classification in contrast to moderate agreement (κ = 0.579; 95% CI 0.525–0.634) for the ER classification system. Diagnostic confidence for intra-articular fracture patterns was superior using the AO/OTA classification compared to ER (*p* < 0.001) with higher confidence agreement (ICC: 0.882 versus 0.831). For extra-articular fractures, ER (κ = 0.817; 95% CI 0.771–0.863) provided better interrater reliability compared to AO/OTA (κ = 0.734; 95% CI 0.692–0.776) with higher diagnostic confidence (*p* < 0.001) and superior agreement between confidence ratings (ICC: 0.881 versus 0.912).

**Conclusions:**

The AO/OTA classification is most suitable to categorize intra-articular scapula fractures with glenoid involvement, whereas the classification system of Euler and Rüedi appears to be superior in extra-articular injury patterns with fractures involving only the scapula body, spine, acromion and coracoid process.

## Background

Accounting for less than 1% of all fractures in humans with trauma history and only 3–5% of traumatic shoulder girdle injuries, scapula fractures are rare. In the absence of predisposing conditions, e.g. malignancy, osteoporosis, or reverse shoulder arthroplasty, fractures of the scapula usually result from high-energy trauma, such as traffic accidents or falls from height. Due to the associated trauma impact and mechanism, scapula fractures are often accompanied by concomitant injuries of the rib cage, spine, ipsilateral clavicle and/or humerus bone. Hence, almost 90% of patients sustaining a scapula fracture display associated bony injuries. In many cases, those fractures are further accompanied by significant soft tissue damage, such as pneumothorax, pulmonary contusion or spinal cord injuries [1–5].

While the incidence of scapula fractures has seemingly increased in recent years, this observed growth may be partly related to the ubiquitous availability of computed tomography (CT) and its use as a primary diagnostic tool in severe accidents, especially in major trauma centers [3]. Fracture morphology and glenoid involvement essentially determine whether a fracture is treated operatively or conservatively. Whilst 80% of the fractures with glenoid involvement are treated operatively, only 9% of body fractures require surgical intervention [6]. Therefore, in patients not receiving CT imaging as primary means of scapula fracture diagnosis, suspicion of glenoid involvement in conventional radiography should lead to subsequent CT imaging to evaluate the necessity of surgical therapy [7]. However, purely extra-articular fractures may also require surgery, primarily depending on the involvement of the glenopolar angle.

Nowadays, plenty of classification systems are available for the categorization of scapula fractures. However, many of those only focus on injury patterns with glenoid involvement, such as the commonly used Ideberg classification [8]. The two most popular classification systems comprising both scapula body and glenoid fractures have been devised by Euler and Rüedi (ER) and through the concerted effort of the “Arbeitsgemeinschaft für Osteosynthesefragen “ (AO) Foundation and the Orthopaedic Trauma Association (OTA) [9, 10]. The ER classification was established in 1996, contains 17 individual fracture types, and allows the combination of various fracture pattern labels [11]. The newer AO/OTA classification of scapula fractures was based on the analysis of 45 CT scans in 2013 and divides scapula fractures in three main groups: scapula process fractures (type A), scapula body fractures (type B) and fractures involving the glenoid fossa (type F). Each superordinate category consists of various subtypes, e.g., glenoid fractures are divided in 11 possible fracture patterns [12]. The applied AO/OTA classification is in accordance with the classification compendium published by the AO in 2018.

In trauma assessment, fracture classification systems serve the main purpose of aligning the language used to characterize certain injury patterns. A good system must cover a wide range of fracture types, have implications on the choice of treatment, and be reproducible by different practitioners. With that being said, the purpose of this study was to compare the AO/OTA and ER classification systems in clinical practice with regard to interobserver reliability and diagnostic confidence. We hypothesized that the AO/OTA system is superior for intra-articular fractures with glenoid involvement, whereas the ER classification may be more suitable for extra-articular scapular body and process fractures.

## Methods

### Study population

**Fig. 1:**
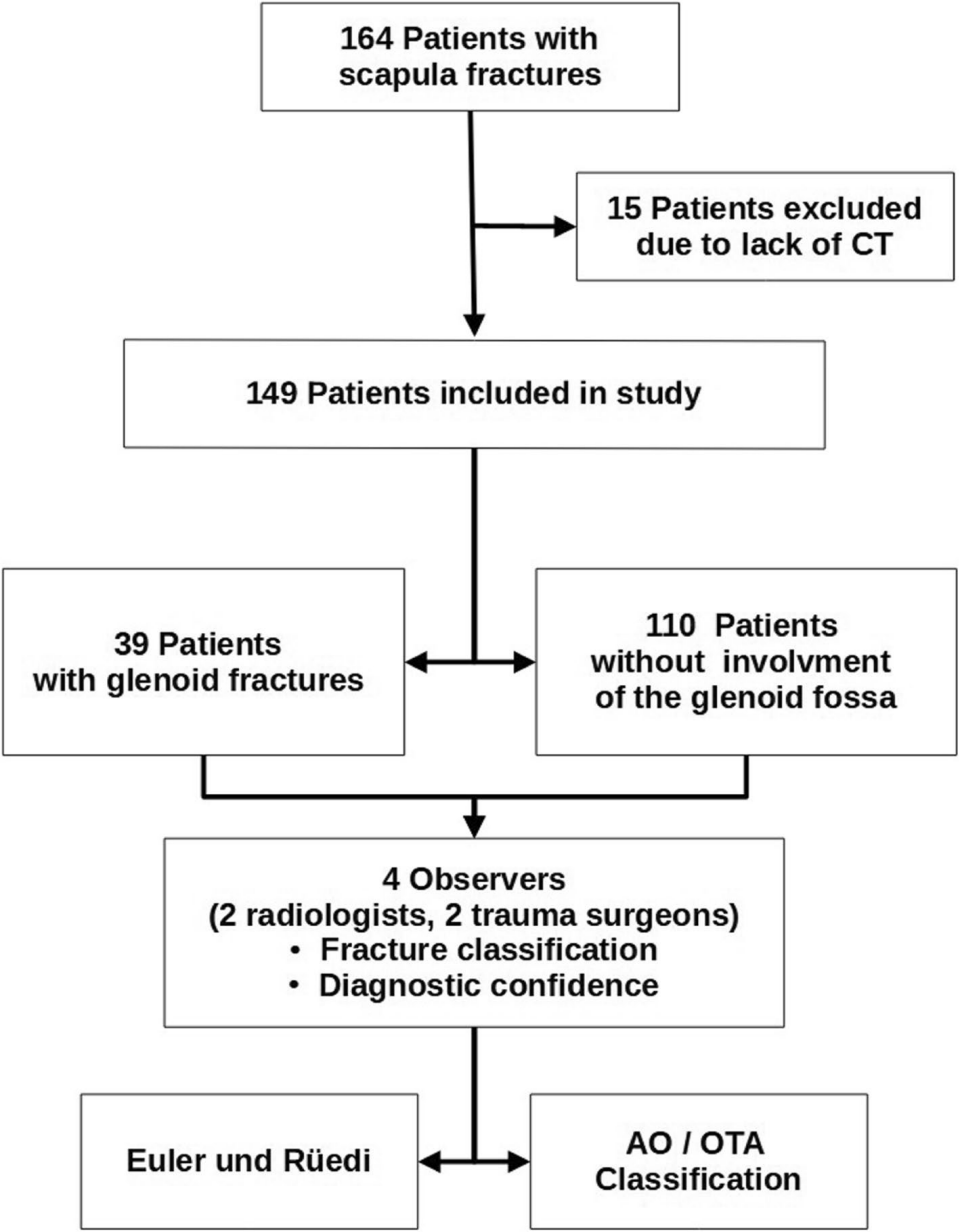
Flow chart for visualization of study structure and patient population

The local institutional review board approved this retrospective investigation and waived the need for additional written informed consent. For this study, the patient records and imaging history of all patients who were treated with any kind of scapula fracture in our level I trauma center at a tertiary-care university hospital between 2010 and 2020 were analyzed. To be eligible for study inclusion, patients had to be at least 18 years of age. Further inclusion criteria included the presence of an acute scapula fracture (time interval between trauma and imaging ≤ 3 weeks) and the availability of CT imaging. Forgoing of CT imaging, lack of orthogonal reconstructions or thin-slice data for free-handed reformatting, and pathologic fractures associated with primary bone tumors or bone metastases were defined as exclusion criteria. The inclusion and exclusion criteria, as well as the resulting study population are summarized in Fig. 1.

### Image analysis

**Fig. 2:**
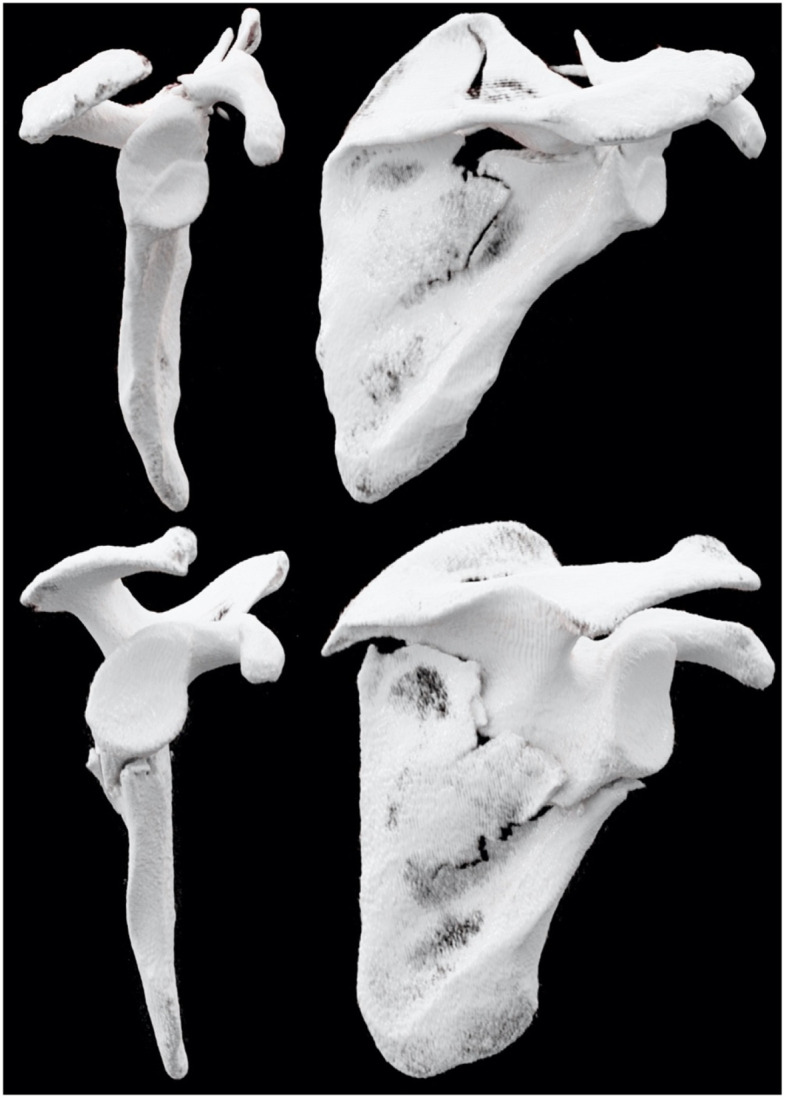
Cinematic volume rendering technique demonstrates complex scapula fractures with intra- und extra-articular injury patterns

In this study, fractures were classified using both the ER (Table 1) and AO/OTA classification systems (Table 2) based on multiplanar reformatting of high-resolution multidetector CT imaging. Two trauma surgeons with 6 (R1) and 4 years (R2) of experience in shoulder surgery and two radiologists with 5 (R3) and 4 years (R4) of musculoskeletal imaging experience evaluated all CT datasets independently using dedicated PACS software (Merlin, Phoenix-PACS, Freiburg, Germany) installed on a standard radiologic workstation with a certified diagnostic monitor (﻿RadiForce RX660, EIZO, Hakusan, Japan). For each patient, observers were provided with orthogonal standard reformations in axial, coronal and sagittal orientation. In addition, 3D volume rendering projections were prepared retrospectively by a radiology resident with five years of clinical experience using a scanner-side workstation with dedicated software (syngo.via, version VB40B, Siemens Healthcare GmbH, Erlangen, Germany) (Fig. 2). During their reads, the observers were provided with handouts illustrating the various fracture patterns of each classification in form of schematic drawings with additional explanation in text form. The four readers did not receive any additional patient information. Particularly, no data on radiological assessment, method of treatment (surgical vs. conservative), and outcome were provided. For each patient, readers were asked for their diagnostic confidence on a five-point Likert scale (5 = total confidence, 4 = high confidence, 3 = moderate confidence, 2 = slight confidence, 1 = little to no confidence).

**Table 1:** Euler and Rüedi scapula fracture classification

A Scapula body fractures	B Scapula process fractures	C Scapula neck fractures	D Articular fractures	E Combined fractures
Isolated or multifragmentary	B1 Spine fracture	C1 Anatomical neck fracture	D1 Glenoid rim fracture	Concomitant humeral head fractures
			D2 Glenoid fossa with a) Inferior glenoid fragment b) Horizontal split fracture c) Coracoglenoid block formation d) Comminuted fractures	
	B2 Coracoid fracture	C2 Surgical neck fracture		
	B3 Acromion fracture	C3 Surgical neck fracture with a)Clavicle fracture b)Ligament tear		
			D3 Scapula neck and body

**Table 2:** AO/OTA scapula fracture classification

A Scapula process fractures	B Scapula body fractures	F Glenoid fossa fractures
A1 Coracoid fracture	B1 Fracture exits the body at ≤ 2 points	F0 Fracture through the extra-articular subchondral bone of the glenoid fossa
		F1.1 Simple, anterior rim fracture
A2 Acromion fracture		F1.2 Posterior rim fracture
	B2 Fracture exits the body at ≥ 3 points	F1.3 Transverse or short oblique fracture
A3 Spine fracture		F2.1 Multifragmentary (≥ 3 articular fragments), glenoid fossa fracture
		F2.2 Multifragmentary (≥ 3 articular fragments with rim exits) fracture with central dislocation

### Statistics

Dedicated software was used for statistical testing (SPSS Statistics Version 28.0, IBM, Armonk, New York, USA). Normal distribution of continuous data was analysed with Kolmogorov–Smirnov tests. Normally distributed data is presented as mean ± standard deviation. For items without normal distribution, we report absolute frequencies and percentages with median values. To measure the interrater reliability for each fracture classification system, Fleiss kappa (κ) was computed with interpretation following Landis and Koch (< 0.00 = poor, 0.00–0.20 = slight, 0.21–0.40 = fair, 0.41–0.60 = moderate, 0.61–0.80 substantial, 0.81–1.00 = almost perfect) [13]. Paired nonparametric variables were compared with Wilcoxon signed rank tests. To estimate the agreement of confidence ratings between observers, the intraclass correlation coefficient (ICC) was calculated based on absolute agreement in a two-way random effects model. ICC results were interpreted in accordance with Koo and Li: < 0.50 = poor, 0.50–0.75 = moderate, 0.75–0.90 = good, > 0.90 = very good [14]. *P* values ≤ 0.05 were considered to indicate statistical significance.

## Results

During the time period of January 2010 – December 2020, the authors identified and reviewed data of 164 patients with documented scapula fractures. Due to a lack of CT imaging or insufficient three-dimensional reformatting, 15 patients were excluded from the study population. Therefore, the final study group consisted of 149 patients, including 21 women (14.09%) and 128 men (85.91%) with a mean age of 47 ± 14 years. The left scapula was fractured in 80 cases (53.69%), the right scapula in 69 (46.31%). Of the 149 patients included, glenoid involvement was ascertained in 39 fracture patterns (26.17%), while 110 cases of extra-articular injuries were recorded (73.83%).

In intra-articular scapula fractures with any form of glenoid involvement, the interrater reliability was substantial (κ = 0.722; 95% confidence interval [CI] 0.676–0.769) for the AO/OTA classification. In contrast, reliability was moderate for the ER classification (κ = 0.579; 95% CI 0.525–0.634; *p* < 0.001). Diagnostic confidence (*p* < 0.001) and the agreement of confidence ratings in fractures with glenoid involvement was higher for the AO/OTA (0.882; 95% CI 0.807–0.933; *p* < 0.001) than for the ER classification (0.831; 95% CI 0.724–0.903; *p* < 0.001). For extra-articular scapula fractures, the calculation of Fleiss kappa suggested almost perfect interrater reliability for the ER classification (κ = 0.817; 95% CI 0.771–0.863; *p* < 0.001), whereas substantial agreement was recorded for the AO/OTA system (κ = 0.734; 95% CI 0.692–0.776; *p* < 0.001). Overall diagnostic confidence (*p* < 0.001) and agreement of confidence ratings between observers was superior for the ER classification (0.912; 95% CI 0.882–0.936; *p* < 0.001) compared to the AO/OTA system (0.881 95% CI 0.840–0.914; *p* < 0.001) when assessing scapula body and process injuries without glenoid involvement. Of note, the interobserver agreement of the two trauma surgeons was higher than the agreement of the two radiologists for both classification systems irrespective of glenoid involvement. Diagnostic confidence ratings in intra- and extra-articular fracture patterns are summarized in Table 3 for both fracture classifications. Of 149 fractures assessed in total, observers deemed 17 injuries (R1 / R2 / R3 / R4: 6 / 6 / 5 / 0) not classifiable with the AO/OTA classification, while 9 fractures patterns (4 / 3 / 1 / 0) were considered not assignable when using the ER classification (Table 4).

**Table 3:** Diagnostic confidence

Diagnostic confidence	Intra-articular fracture ratings (*n* = 156)		Extra-articular fracture ratings (*n* = 440)	
	AO/OTA	ER	AO/OTA	ER
**5**	85 (54,49%)	41 (26,28%)	262 (59,55%)	420 (95,45%)
**4**	36 (23,07%)	64 (41,03%)	143 (32,50%)	14 (2,73%)
**3**	22 (14.10%)	36 (23,08%)	15 (3,41%)	1 (0,23%)
**2**	8 (5.13%)	6 (3,85%)	3 (0,68%)	0 (0%)
**1**	5 (3,21%)	9 (5,77%)	17 (3,86%)	5 (1,14%)
**MEDIAN (IQR)**	5 (4 – 5)	4 (3 – 5)	5 (4 – 5)	5 (5 – 5)

*AO/OTA* AO Foundation / Orthopaedic Trauma Association classification, *ER* Euler and Rüedi classification, *IQR* interquartile range (25% – 75%)

**Table 4:** Not classifiable fractures

Fractures deemed not classifiable	Intra-articular fracture ratings (*n* = 156)	Extra-articular fracture ratings (*n* = 440)
AO/OTA	0 (0%)	17 (3.86%)
ER	5 (3.21%)	4 (0.91%)

*AO/OTA* AO Foundation / Orthopaedic Trauma Association classification, *ER* Euler and Rüedi classification

## Discussion

In this study, we compared the interrater reliability and diagnostic confidence of four observers (two trauma surgeons, two radiologists), who used both the ER and AO/OTA classification to classify 149 scapula fractures on the basis of high-resolution multidetector CT datasets. Our results suggest superiority of the AO/OTA system in categorizing intra-articular fractures with glenoid involvement, whereas the ER classification appears to be more suitable for the grading of extra-articular scapula fractures that do not involve the glenoid fossa. Since scapula fractures represent rare injuries, we aimed to analyze the usability for each fracture classification in clinical routine.

The majority of injuries in our population occurred in young (mean age of 47 years) and male patients (85.91%), which is concordant with the existing literature. This finding may be attributed to the association of scapula fractures with high-energy traumas, which are less common in females and elderly patients. Our quantitative results for fracture classification applicability are in line with previous studies favoring the AO/OTA system in the presence of glenoid involvement: Ter Meulen et al. used quantitative three-dimensional CT analysis with fracture pattern heat maps to demonstrate that the AO/OTA classification is capable of adequately discriminating glenoid fracture patterns [15]. Gilbert et al.⁠. also reported superiority of the AO/OTA classification in case of glenoid involvement in a study of 84 glenoid fractures [16]. On the other hand, Neuhaus et al. stated that using the AO/OTA classification for extra-articular scapula body and process fractures generated more interrater disagreement than the application for intra-articular fractures, confirming our findings that the AO/OTA classification is inferior for extra-articular fracture patterns [17]. Bartoníček et al. questioned the practical relevance of the AO/OTA classification due to its primary focus on the course of fracture lines in the glenoid fossa [18]. In our study, 17 scapula fractures in total were categorized as “not classifiable” using the AO/OTA system, whereas only 9 fracture patterns received that distinction when applying the ER classification. Of these injuries, all that were deemed unclassifiable with the AO/OTA system demonstrated an extra-articular fracture pattern, supporting our assumption that the AO/OTA classification offers a suitable class for most glenoid fractures. Despite our findings in extra-articular patterns, it must be stated that the AO/OTA classification, although not as good as the ER system, still provided substantial agreement and high consistency in confidence reports for fractures without glenoid involvement.

With approximately 45% of scapula fractures involving the scapula body, it is important to note that the majority of extra-articular scapula fractures are treated conservatively; however, surgical intervention is advocated for severely displaced fracture patterns, particularly for those involving the lateral column [12]. In exclusively extra-articular scapula fractures, the most relevant information for the choice of treatment are shortening or significant involvement of the glenopolar angle [9]. These factors should be considered in the design of a unified classification system that is reliable and clinically applicable for both intra- and extra-articular scapula fractures, hence allowing for treatment decisions with good functional outcome. Since the current version of the AO/OTA classification with focus on the glenoid fossa appears to be reliable for intra-articular fractures but imperfect for extra-articular patterns, Audigé et al. have attempted to amplify the AO/OTA system in 2014 by placing more emphasis on the affected scapula body margins; however, the proposed classification has not found wide range application in clinical practice due to its rather theoretical character [12].

Our study includes several limitations. First, all scapula fractures were analyzed in close temporal proximity to the causative trauma without clinical and radiological follow-up. Therefore, the impact of fracture classifications on the patient outcome cannot be thoroughly assessed. Second, since the reading time required by each observer for the fracture classification process was not measured, the data presented in this work are limited to the applicability of each classification for different injury patterns without information on the practicability in clinical routine.

In contrast, the strengths of this study lie in the high number of patients and the interdisciplinary observer team, that can be considered as representative of a real-world scenario, where radiologists and trauma surgeons work in close collaboration to facilitate the optimal treatment of injured patients.

## Conclusion

The AO/OTA classification is most suitable to categorize intra-articular scapula fractures with glenoid involvement, whereas the classification system of Euler and Rüedi appears to be superior in extra-articular injury patterns. While none of the available systems seems to provide an entirely satisfying classification process for all scapula fractures, we believe that an optimized system, which integrates the best components of both classifications, would positively influence the diagnostic assessment and therapy planning in patients with scapula injuries. Adhering to one unified system would also be beneficial for the interdisciplinary communication between radiologists and trauma surgeons, leading to better clinical outcome in the process.

## Data Availability

The datasets generated and/or analyzed during this study are not publicly available as CT data and DICOM headers contain patient information. Data can be obtained on reasonable request from the corresponding author.

## Abbreviations

AO: “Arbeitsgemeinschaft für Osteosynthesefragen” [German]
CI: Confidence interval
ICC: Intraclass correlation coefficient
OTA: Orthopaedic Trauma Association
